# Cost-effectiveness of Osimertinib in activating epidermal growth factor receptor gene (*EGFR*)-mutations in first-line for advanced non-small cell lung cancer

**DOI:** 10.20517/cdr.2021.14

**Published:** 2021-04-27

**Authors:** Jacopo Giuliani, Andrea Bonetti

**Affiliations:** Department of Oncology, Mater Salutis Hospital - Az. ULSS 9 Scaligera, Legnago (VR) 1-37045, Italy.

**Keywords:** NSCLC, activating *EGFR*-mutations, first-line treatment, TKIs, cost of drugs

The introduction of first- or second-generation epidermal growth factor receptor gene (*EGFR*) tyrosine kinase inhibitors (TKIs) in chemonaive patients with advanced non-small cell lung cancer (NSCLC) has radically changed the treatment in this molecular subgroup, with an improvement in progression-free survival (PFS) compared to standard chemotherapy^[1]^. The introduction of these active new agents is associated with a relevant increase of costs, and the most fitting example is represented by the recent introduction of osimertinib in this setting. In fact, the topic of costs has become preponderant in oncology and radiotherapy, as well as in relation to the introduction of target biological agents and immunotherapy, with their greatest budgetary impact^[2]^. Recently, Aguilar-Serra *et al.*^[3]^ performed a cost-effectiveness analysis of osimertinib *vs.* standard first-line TKIs (erlotinib and gefitinib) in advanced NSCLC. They concluded that osimertinib was more effective in terms of quality-adjusted life-years (QALYs) gained than comparators (erlotinib-gefitinib), but a discount greater than 60% in osimertinib acquisition cost would be required to obtain a cost-effective alternative.

The aim of our study was to assess the pharmacological costs of TKIs (erlotinib, gefitinib, afatinib and osimertinib) in patients with activating *EGFR *mutations in first-line treatment for advanced NSCLC. Pivotal phase III randomized controlled trials (RCTs) were considered. The last available update of each trial was considered as the original source. The deadline for trial publication and/or presentation was 30 June 2020. Incremental cost-effectiveness ratio (ICER) was calculated as the ratio between the difference of the costs in the intervention and in the control groups (pharmacy costs) and the difference between the effect in the intervention and in the control groups [overall survival (OS)]. The costs of drugs were based on those at the pharmacy of our hospital and are expressed in euros (€), updated to June 2020. The pharmacy costs of drugs are summarized on Table 1. The dosages of drugs were considered according to those reported in each RCT. The European Society for Medical Oncology Magnitude of Clinical Benefit Scale (ESMO-MCBS) was applied the to the pivotal RCTs^[4] ^to derive a relative ranking of clinical benefit^[5]^. All data were reviewed by two investigators (Giuliani J and Bonetti A) and separately computed by two investigators (Giuliani J and Bonetti A).

**Table 1:** Cost of drugs

**Drug**	**Pharmacy cost (€) (dose)**
Gemcitabine	9.90 (1000 mg)
Paclitaxel	6.19 (100 mg)
Docetaxel	9.84 (80 mg)
Carboplatin	7.18 (150 mg)
Cisplatin	8.80 (50 mg)
Vinorelbine	6.54 (10 mg)
Nab-paclitaxel	214.48 (100 mg)
Bevacizumab	2004.61 (1 administration at 7.5 mg pro Kg) 2672.28 (1 administration at 15.0 mg pro Kg)
Pemetrexed	226.65 (100 mg)
Pembrolizumab	2056.08 (100 mg)
Erlotinib	45.80 (150 mg tablet)
Gefitinib	72.06 (250 mg tablet)
Afatinib	65.85 (20 mg tablet)
Osimertinib	145.28 (80 mg tablet)

Nine phase III RCTs^[6-14]^, including 2291 patients, were considered. The OS of TKIs ranged from 18.8 months for gefitinib in the IPASS trial^[8,15]^ to 38.6 months in the FLAURA trial^[14,16]^. ESMO-MCBS reached Grade 4 for OPTIMAL trial^[6]^, EURTAC trial^[7]^, IPASS trial^[8]^, LUX-Lung 3 trial^[12]^ and FLAURA trial^[14]^; Grade 3 for NEJ2002 trial^[9]^, WJTOG3405 trial^[10]^ and LUX-Lung 6^[13]^; and Grade 1 for First-SIGNAL trial^[11]^. The lowest cost for 1 month of OS gain was associated with osimertinib, at €9740 per month OS gained [Table 2].

**Table 2:** Pharmacological costs and difference in OS with the different treatment regimens of the pivotal phase III RCTs in first-line treatment for advanced NSCLC with activating EGFR mutations

**Ref./Trial**	**Comparative regimens**	***N*** ** of patients**	**OS (months)**	***P*** **-value**	**Difference in OS (months)**	**Median duration of treatment (months) **	**Costs of therapy (€)**	**Difference in costs (€)**	**ICER (€)**
Zhou *et al.*^[6]^ OPTIMAL	Carboplatin + gemcitabine	72	27.2^a^	NS^a^	-4.4	2.4	246	17,576	NA
	Erlotinib	82	22.8^a^			12.8	17,822		
Rosell *et al.*^[7]^ EURTAC	Cisplatin + docetaxel/gemcitabine	87	22.1^b^	NS^b^	0.8	2.8	238-222	11,179-11,195	13,974-13,994
	Erlotinib	86	22.9^b^			8.2	11,417		
Mok *et al.*^[8]^ IPASS	Carboplatin + paclitaxel	129	17.4^c^	NS^c^	1.4	3.4	202	13,818	9870
	Gefitinib	132	18.8^c^			6.4	14,020		
Maemondo *et al.*^[9]^ NEJ2002	Carboplatin + paclitaxel	110	26.6^d^	NS^d^	1.1	2.8	202	21,923	19,930
	Gefitinib	114	27.7^d^			10.1	22,125		
Mitsudomi *et al.*^[10]^ WJTOG3405	Cisplatin + docetaxel	86	37.3^e^	NS^e^	-2.4	2.1	238	11,591	NA
	Gefitinib	86	34.9^e^			5.4	11,829		
Han *et al.*^[11]^ First-SIGNAL	Cisplatin + gemcitabine	26	22.9	NS	-0.6	4.1	334	11,799	NA
	Gefitinib	22	22.3			5.4	11,829		
Sequist *et al.*^[12]^ LUX-Lung 3	Cisplatin + pemetrexed	111	28.2^f^	NS^f^	0.0	4.1	13,051	30,989	NA
	Afatinib	229	28.2^f^			11.0	44,040		
Wu *et al.*^[13]^ LUX-Lung 6	Cisplatin + gemcitabine	122	23.5^f^	NS^f^	-0.4	2.9	238	52,210	NA
	Afatinib	242	23.1^f^			13.1	52,448		
Soria *et al.*^[14]^ FLAURA	Standard EGFR-TKI^g^	277	31.8^h^	0.046^h^	6.8	11.5	16,012-25,192	66,230-75,410	9740-11,090
	Osimertinib	279	38.6^h^			20.7	91,422		

^a^Update on OS^[17]^. ^b^Update on OS^[18]^. ^c^Update on OS^[15]^. ^d^Update on OS^[19]^. ^e^Update on OS^[20]^. ^f^Update on OS^[21]^. ^g^Gefitinib or erlotinib. ^h^Update on OS^[16]^. RCTs: Randomized controlled trials; EGFR: epidermal growth factor receptor; NSCLC: non-small cell lung cancer; N: number; OS: overall survival; ICER: incremental cost-effectiveness ratio [expressed as the difference (€) per month - OS gained]; NS: not significant; NA: not applicable.

Two main variables influence pharmacy costs: the efficacy of treatment and the price of drugs. The first variable is related to the patient’s inclusions criteria, and we know that results from RCTs might not be representative of daily clinical practice (i.e., of patients treated outside such trials). The price of drugs is the second strong variable. In fact, there may be a cost standardization problem within different European countries (in Italy, there are no significant pharmacy cost differences among the different regions), due to the use of local pharmacy cost. Another limit is related to the consideration of only direct costs (which account for about 55% of total medical expenses). In Europe, expenditure for cancer drugs amounted to €10 billion in 2005, increasing more than three times to €32 billion in 2018^[17]^. In this scenario, European countries negotiate the price of new drugs with the manufacturers with the aim to obtain a discount, so as to allow more patients to be treated. This results in “confidential rebates” (i.e., not publicly available), which may hamper access to drugs with a consequent overpayment without improving the value of drugs. The extraordinary costs of novel treatments may form a new type of resistance, costs resistance. In several countries, this may preclude treatments with these compounds.

There are several published articles, mostly in China, regarding this topic. However, to our knowledge, this is the first cost-effectiveness analysis of TKIs in patients with activating *EGFR*-mutations in first-line treatment for advanced NSCLC in Europe.

In addition, the annual cost of drugs treatment (€116,880 for osimertinib, €118,840 for gefitinib and €165,528 for erlotinib) are not in line with those reported in the literature, which indicate implementing intervention for thresholds of less than $61,500 (€57,138) per life-year gained^[18]^.

We also compared the pharmacy costs of TKIs (osimertinib, erlotinib, gefitinib and afatinib) with the pharmacy costs of other immune check-point inhibitors (ICIs), such as nivolumab, pembrolizumab and atezolizumab, registered in other tumors (e.g., NSCLC, head and neck carcinoma and urological malignancies) and known as the most expensive new drugs in medical oncology^[19-24]^, as well as the costs of the reference elements in international markets, 18 karat (K) gold and platinum. All TKIs have the highest cost per gram, with €305.33 for erlotinib, €288.24 for gefitinib, €3292.50 for afatinib and €1816.00 for osimertinib, with a Δ toward 18 K gold and platinum per gram of €258.43 and €283.88 for erlotinib, respectively; €241.34 and €266.79 for gefitinib, respectively; €3245.60 and €3271.05 for afatinib, respectively; and €1769.10 and €1794.55 for osimertinib, respectively. This leads us to think that ICIs are not the most expensive targeted agents, but there are other more expensive ones. Thus, there is no doubt that data on osimertinib are good in daily clinical practice^[11,13]^, but a reduction in pharmacological costs is mandatory if we want to consider TKIs (in particular, osimertinib) more advantageous in terms of cost-effectiveness.

In conclusion, based on ICER, osimertinib is more cost-effective than the other TKIs (erlotinib, gefitinib and afatinib) in patients with activating *EGFR *mutations in first-line treatment for advanced NSCLC. The data on osimertinib are good in daily clinical practice (also confirmed by the high grade of clinical benefit on ESMO scale^[3]^), but a reduction in pharmacological costs is mandatory if we want to consider osimertinib more cost-effective in first-line treatment for *EGFR*-mutated advanced NSCLC.

## DECLARATIONS

### Copyright

© The Author(s) 2021.
